# Shape of the Urinary Bladder

**Published:** 1905-11

**Authors:** 


### ABSTRACTS.

### Shape of the Urinary Bladder.

VOELCKER AND LICHTENBERG publish from Czerny's Surgical Clinic at Heidelberg (*Münch. Med. Wochenschrift,* August 15, 1905) a paper on the shape of the urinary bladder as revealed by skiagraphy. The ordinary means of examination as weli as cystoscopy give but little information in regard to the shape of the organ. The soluble bismuth compounds are too poisonous to inject into the bladder for the purpose of rendering it opaque, and the insoluble ones are liable to leave material that may form the nucleus of calculi. A 2 per cent. collargolum solution is, however, eminently adapted for the purpose, for it does not irritate the vesical mucosa. On the contrary, in some cases of chronic cystitis there was marked improvement after collargolum injection. Voelcker and Lichtenberg have made extensive use of this accidental discovery, as its injection causes no pain whatever, and employ it in cystitis exclusively in place of other antiseptics, such as silver nitrate which is notoriously painful. In chronic cystitis from prostatic hypertrophy, for instance, they inject 3½ ounces of a 1 per cent. collargolum solution, which may be left in the bladder as long as desired, and even permanently.

Excellent skiagraphs were obtained, giving valuable information in prostatic hypertrophy, vesical prolapse in the female, and other displacements and deformities of the organ. The article is illustrated.

